# Ants Sow the Seeds of Global Diversification in Flowering Plants

**DOI:** 10.1371/journal.pone.0005480

**Published:** 2009-05-13

**Authors:** Szabolcs Lengyel, Aaron D. Gove, Andrew M. Latimer, Jonathan D. Majer, Robert R. Dunn

**Affiliations:** 1 Department of Biology, North Carolina State University, Raleigh, North Carolina, United States of America; 2 Department of Ecology, University of Debrecen, Debrecen, Hungary; 3 Centre for Ecosystem Diversity and Dynamics, Curtin University of Technology, Perth, Australia; 4 Department of Plant Sciences, University of California Davis, Davis, California, United States of America; Centre National de la Recherche Scientifique, France

## Abstract

**Background:**

The extraordinary diversification of angiosperm plants in the Cretaceous and Tertiary periods has produced an estimated 250,000–300,000 living angiosperm species and has fundamentally altered terrestrial ecosystems. Interactions with animals as pollinators or seed dispersers have long been suspected as drivers of angiosperm diversification, yet empirical examples remain sparse or inconclusive. Seed dispersal by ants (myrmecochory) may drive diversification as it can reduce extinction by providing selective advantages to plants and can increase speciation by enhancing geographical isolation by extremely limited dispersal distances.

**Methodology/Principal Findings:**

Using the most comprehensive sister-group comparison to date, we tested the hypothesis that myrmecochory leads to higher diversification rates in angiosperm plants. As predicted, diversification rates were substantially higher in ant-dispersed plants than in their non-myrmecochorous relatives. Data from 101 angiosperm lineages in 241 genera from all continents except Antarctica revealed that ant-dispersed lineages contained on average more than twice as many species as did their non-myrmecochorous sister groups. Contrasts in species diversity between sister groups demonstrated that diversification rates did not depend on seed dispersal mode in the sister group and were higher in myrmecochorous lineages in most biogeographic regions.

**Conclusions/Significance:**

Myrmecochory, which has evolved independently at least 100 times in angiosperms and is estimated to be present in at least 77 families and 11 000 species, is a key evolutionary innovation and a globally important driver of plant diversity. Myrmecochory provides the best example to date for a consistent effect of any mutualism on large-scale diversification.

## Introduction

The diversification of angiosperm plants is one of the greatest terrestrial radiations on Earth. Despite considerable progress in fields as varied as paleobiology, phylogenetics, evolutionary developmental biology and genetics [1], much about the origin and diversification of angiosperms is still largely a mystery [2]. One particularly challenging task is to explain the shifts in diversification rates that have produced differences in species diversity spanning several orders of magnitude between sister lineages [3].

Interactions with animals as pollinators or seed dispersers have long been suspected as drivers of angiosperm diversification [4]–[6] and theory shows that such interspecific interactions could drive diversification by influencing rates of both speciation [7] and extinction [8]. Recent evidence suggests that insect pollinators were instrumental in the early radiation of angiosperms [9], whereas other interactions, such as seed dispersal mutualisms, were hypothesized to sustain high diversification rates in later Tertiary radiations [10]. Seed dispersal fundamentally influences plant recruitment, colonization ability and population persistence. Seed dispersal mutualisms with animals provide plants with a reliable dispersing agent, which may increase plant fitness and population growth rate, and may thus reduce extinction rates [11], [12]. Moreover, seed dispersal mutualisms may also limit dispersal distances, which may reduce gene flow among populations, facilitate local adaptations and may thus increase speciation rates in plants [13]. Empirical studies comparing vertebrate-dispersed and non-animal dispersed plants, however, found no effect of animal dispersal on diversification [14]–[17], or found an effect only for certain growth forms such as woody plants [12], [18] or tropical understorey herbs [19]. Such equivocal results could indicate that the effect of seed dispersal mutualisms on diversification varies by either the ancestral mode of seed dispersal, biogeographic region, or the identity of the animal partner and the distance it moves seeds.

Compared to seed dispersal mediated by vertebrates, seed dispersal by ants (myrmecochory) is associated with extremely short distances (average: 1 m, range: 0.01–77 m) [20]. If seed dispersal mutualisms are associated with limited dispersal distances, the impact of such mutualisms on diversification via increased isolation and speciation should be more pronounced in myrmecochorous plants than in plants dispersed by vertebrates. In addition, seed dispersal mutualisms with ants provide myrmecochorous plants with several selective advantages that are not present in other dispersal modes (reviewed in [21]–[23]). These benefits can increase plant fitness and population growth rate, thereby reducing extinction risks. Myrmecochorous plants, therefore, can be predicted to diversify faster than plants with other dispersal modes through higher speciation rates, reduced extinction rates or both.

We tested the hypothesis that the evolutionary transitions to myrmecochory lead to higher diversification in angiosperm plants using sister-group comparisons. We first compiled an updated, referenced, globally comprehensive list of myrmecochorous genera to represent the global distribution of myrmecochory in angiosperms (**Table S1**, [24]). We used these data to test whether there is an overall effect of higher diversification in myrmecochorous lineages compared to their sister lineages. We also tested whether differences in diversification rates are contingent on biogeography or the likely ancestral dispersal mode in the sister group using general linear models analyzing contrasts in species diversity. Sister-group comparisons are one of the best methods to test differences in diversification rates because this method is robust to differences in evolutionary age (by definition, sister groups are of the same age) and to non-independence of taxa, and it minimizes the effect of confounding variables (sister groups differ only in traits evolved since the last common ancestor) and other noise that may obscure relationships between evolutionary variables [25].

**Figure 1:**
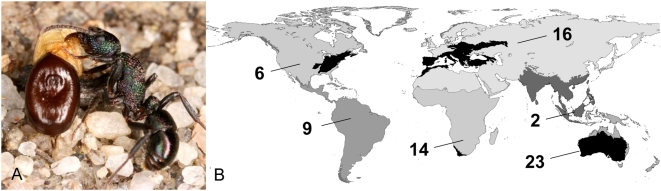
Convergent evolution of elaiosomes as an adaptation for seed dispersal by ants (myrmecochory) in angiosperm plants. (A) *Rhytidoponera metallica* ant holding a seed of *Acacia neurophylla* by the elaiosome during seed transport [Photograph by Benoit Guenard]. (B) Myrmecochore diversity hotspots (in black) and number of myrmecochorous plant lineages in major biogeographic regions (in shades of grey). Lineages distributed in more than one region (not shown) are Holarctic (n = 14), Old World (n = 5), pan-tropical (n = 2), or worldwide (n = 10).

The signal adaptation for myrmecochory is the elaiosome, a lipid-rich, N-poor appendage of the seed (**Fig. 1A**). Elaiosomes attract ants and elicit the transport of the seeds by the ants into their nest. In the nest, the elaiosome is removed and eaten or fed to the larvae, and the seed is deposited in an underground nest chamber or in a refuse pile outside the nest. Elaiosomes thus function as rewards for the ants [26], [27]. Myrmecochory has been reported in 3000 species and in over 80 families of plants [21], [26]. Myrmecochorous plants make up 30–40% of herbs in eastern North American forests [26], [28], 40–50% of herbs in eastern European deciduous forests [22], 1200 species or 20% of plant species in South African fynbos [29], and 134 genera and no fewer than 1500 species in Australia [30], [31]. Recent advances in phylogenetic systematics make it possible, for the first time, to address large-scale patterns in the evolutionary origins of ant-plant seed dispersal mutualisms in angiosperms [32]. Our study is the most comprehensive analysis to date of the role of a mutualism with animals in plant diversification and the first to make comparisons relative to biogeographic regions and dispersal mode of the sister group (see Materials and Methods at the end).

## Results

We found that seed dispersal by ants has evolved independently in at least 101 lineages consisting of 241 genera in 55 angiosperm families (**Table S1**). Three or more origins of myrmecochory were from six families: Asteraceae (10 origins), Euphorbiaceae (8), Fabaceae (6), Ranunculaceae (5), Lamiaceae (4), and Liliaceae (3). Two origins were found in 16 families and one in 33 families. Our conservative estimate of the number of plant species in which seeds are dispersed by ants is 11,532 species or c. 4.5% of all known angiosperm species (**Table S1**, [24]).

As previously suggested [26], [31], most origins of myrmecochory were found in temperate or dry mediterranean areas of Australia, South Africa (almost exclusively from the Cape Floristic Region, CFR) and the Palearctic and Nearctic regions (**Fig. 1B**). We found no evidence that myrmecochorous lineages tend to be restricted geographically: 31% of the myrmecochorous lineages (n = 101), showed multi-regional, pan-tropical or world-wide distributions (**Fig. 1**).

Myrmecochorous lineages had more species than their sister group in 68 of the 101 comparisons with complete data. This proportion differed significantly from chance (sign-test, *P* = 0.0006). The mean value of the species diversity contrast (difference in log-transformed species numbers) was 0.35±0.83 (S.D.) (range: −1.89 and 2.18), which differed significantly from 0, the value expected under the null hypothesis of no difference between the lineages (95% CI: 0.19–0.52; *t*_97_ = 4.258, *P*<0.0001). Myrmecochorous lineages contained more than twice the number of species in non-myrmecochorous lineages (10^0.35^ ≈ 2.24). The magnitude of the differences was greater when the myrmecochorous lineage had more species (mean contrast: 0.80±0.57 (S.D.), factor of >6) than in cases when the non-myrmecochorous lineage had more species (0.56±0.44 (S.D.), factor of 3.6).

**Figure 2:**
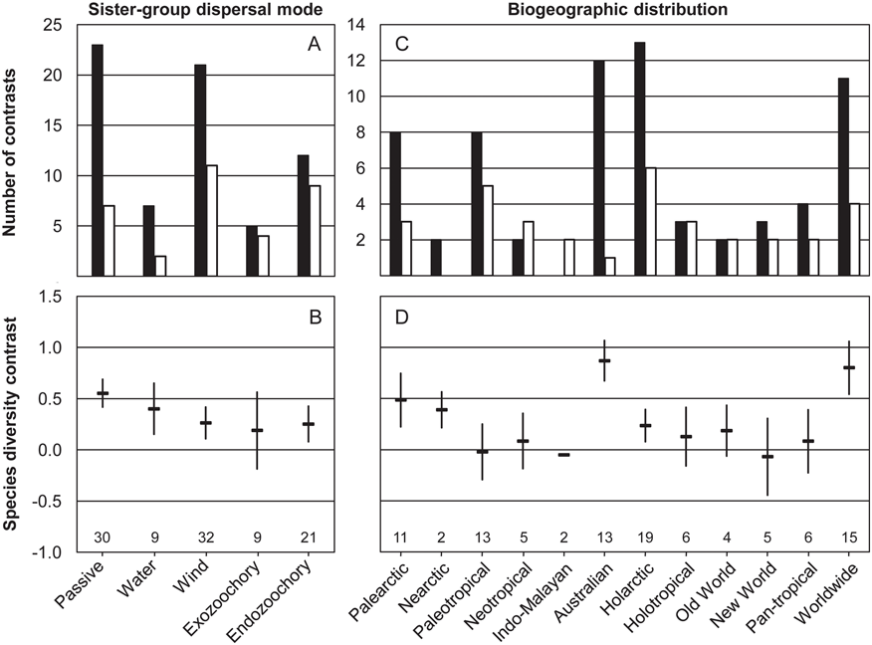
Diversification in sister lineages of myrmecochorous and non-myrmecochorous plants by seed dispersal mode in the sister group (A–B) and by biogeographic region (C–D). Top panels show the number of contrasts in which the mymecochorous lineage is more diverse (closed bars), or when the non-myrmecochorous lineage is more diverse (open bars). Bottom panels show species diversity contrasts (difference in log-transformed species numbers; mean±1 S.E.) to illustrate the magnitude of differences between sister lineages, with positive values indicating more species in the myrmecochorous lineage than in its sister group. The number of contrasts is shown above the X axis in bottom panels. General linear mixed models with sister-group dispersal mode (fixed effect) and distribution type (random effect) showed that dispersal mode did not influence either the direction (Model 1: logistic regression, *F*_4,85_ = 0.751, *P* = 0.560) or the magnitude of contrasts (Model 2: *F*_4,85_ = 0.756, *P* = 0.557). The evaluation of the random effect of biogeographic region by comparing intercept S.D. to residual S.D. [64] showed that variation by biogeographic distribution type also was not influential (Model 1: intercept S.D.: 0.024<residual S.D.: 0.999; Model 2: intercept S.D.: 0.249<residual S.D.: 0.798).

The higher diversification rates in myrmecochorous lineages relative to their sister groups did not depend on seed dispersal mode in the sister group and did not vary significantly by biogeographic region (**Fig. 2**). Most contrasts supported the hypothesis and mean species diversity contrasts were positive (myrmecochores>non-myrmecochores) in all sister-group dispersal modes (**Fig. 2A, B**). Myrmecochorous lineages had consistently more species than did their sister lineages in 9 of 12 biogeographic distribution types, particularly in regions with many origins (Australia, Palearctic) and in widespread distribution types (Holarctic, Old World, worldwide) as shown by positive mean contrasts (**Fig. 2C, D**). Myrmecochorous lineages and their sister groups on average held equal numbers of species (species diversity contrast≈0) in comparisons from the Paleotropics (mainly South African CFR), and most tropical regions (Neotropical/New World, Holotropical, Pan-tropical).

## Discussion

Our results show that the evolution of a seed dispersal mutualism, myrmecochory, is consistently associated with accelerated diversification in angiosperm plants. Our results are the strongest evidence to date for a consistent and strong effect of a species interaction (here myrmecochory) on diversification rates in angiosperms. The effect of myrmecochory on diversification appears independent of the dispersal mode of the sister group and biogeographic region. The evolution of elaiosomes, a simple trait that facilitates seed dispersal mutualisms with ants, appears to have consequences for speciation and extinction besides providing fitness benefits to plants. Elaiosomes thus provide an example for the repeated, convergent evolution of a key innovation [33] that leads to increased diversification similarly in a wide range of plant lineages.

Our study raises the known number of independent evolutionary origins of myrmecochory to above 100 and the probable number of myrmecochorous species from 3000 [26], [30] to above 11,000 or >4.5% of all known angiosperm species. However, our study still substantially underestimates the real numbers of elaiosome origins and myrmecochorous species. First, even in some of the groups included here, there were multiple origins of elaiosomes. For example, at least six origins are known in Polygalaceae [34], of which only one (the earliest) was considered here. Second, our estimate excludes several large genera in which myrmecochory is well-known but in which the exact number of myrmecochorous species was not known or for which sub-generic phylogenies were not available (e.g., *Acacia*, *Acalypha*, *Calathea*, *Carex*, *Cleome*, *Cytisus*, *Globba*, *Hepatica*, *Iris*, *Jatropha*, *Peperomia*, *Potentilla*, *Primula*, c. 8500 species), or in tropical lineages in which myrmecochory is part of a more complex plant-ant mutualism (ant-gardens in e.g., *Aechmea*, *Anthurium*, *Hoya*, *Philodendron*, c. 2000 species).

With at least 100, but possibly as many as 140+ independent origins ([24]), elaiosomes have evolved convergently and independently more often than any other morphological trait related to a mutualism yet studied. In part, the frequency of origins may reflect the relatively low costs of evolving a small, fleshy seed appendage and the consistent benefits that accrue to plants in which elaiosomes evolve. Once it has evolved, myrmecochory may facilitate diversification in two ways [31]. First, myrmecochory can increase plant fitness by enhancing seed survival or germination success, hence, it may lead to reduced extinction rates. Seeds are often moved by ants to microsites that are safe from predators, rich in nutrients, and are suitable for long-term storage and survival during environmental stress [21], [23]. These benefits, which do not typically exist in other dispersal modes, may be especially important in unpredictable, nutrient-limited, or inhospitable (open, dry, fire-prone) environments, such as mediterranean-climate shrub vegetation [30]. Even though ants disperse seeds short distances, they also mix them, which may reduce competition among parent plants and kin [35]. Reduced competition, along with the avoidance of seed predators, may be especially important in closed environments, such as northern temperate forests. Second, myrmecochory can reduce gene flow among spatially distinct populations. Recent evidence from genetic studies shows that limited seed dispersal in myrmecochory can lead to strong genetic structure within populations even at spatial scales as small as few meters [36], [37]. The failure of myrmecochores to maintain gene flow across barriers may lead to reproductive isolation of sub-populations, which may facilitate speciation. These patterns suggest that in myrmecochorous plants, isolated sub-populations can exist in small geographic areas and that, remarkably, these small sub-populations can still have high local population fitness, reduced rates of extinction and higher probabilities for speciation.

It is important to note that although myrmecochory may reduce gene flow among distant populations, it does not prevent it [38]. Recent observations suggest that seeds of myrmecochorous plants are occasionally dispersed by non-standard dispersal agents such as deer and emus [39], [40]. Seeds of myrmecochorous plants, therefore, may occasionally disperse across barriers, but once the new habitat is colonized, most gene flow is likely to be local, thereby facilitating speciation. Because myrmecochory provides various selective benefits to plants and ants are present in most ecosystems, myrmecochory may even pre-adapt plants to successfully colonize new habitats once dispersal over barriers occurs [41]. This effect may explain why many invasive plants are myrmecochorous [42], [43].

In conclusion, our results show that transitions to myrmecochory through the convergent evolution of elaiosomes played an important role in the diversification of angiosperms, giving rise to lineages currently with c. 11,000 species, more than four percent of angiosperm species diversity. Myrmecochory may decrease extinction rates by increasing plant fitness and enhance speciation rates by reducing gene flow through short dispersal distances. These potential properties of myrmecochory, in combination with random and infrequent long-distance dispersal effected by non-standard dispersers, may explain the high proportion and importance of myrmecochorous plants in various communities and habitat types across the Earth. Our results thus suggest that myrmecochory is a globally important, regionally major driver of angiosperm diversity.

## Materials and Methods

**Dataset.** We compiled a list of myrmecochorous plant genera from various literature sources (books, regional floras, reviews, primary literature; please see references in **Table S1**). We identified plants as myrmecochorous if their seeds had elaiosomes, the signal adaptation for seed dispersal by ants [26]. We estimate that our complete dataset (334 genera from 77 families, [24]) contains information on at least 90% of the genera in which elaiosome-bearing seeds have been reported to date (possibly over 350 genera). The dataset used in this study is a subset of the complete dataset, which includes all lineages for which all relevant information (phylogeny, species number, sister-group seed dispersal mode, biogeographical distribution) was available.

### Phylogenies and sister groups

We identified independent evolutionary origins of myrmecochory using recent phylogenetic reconstructions. Because a genus-level angiosperm supertree does not exist, we were not able to map myrmecochory directly on one phylogenetic tree to identify independent origins. Instead, we used information on the sister group and the next higher node (see later). We sought phylogenetic hypotheses based on molecular data, which had sufficient sampling to allow the identification of the node where transition to myrmecochory occurred. A lineage (monophyletic genus or genera) was identified as myrmecochorous if the majority (>50%) of the constituent species in which dispersal mode was known had elaiosome-bearing seeds. This definition had to be applied in only a few cases, though, because in most lineages, all species of known dispersal mode were myrmecochorous. The inclusion or exclusion of these “mixed” myrmecochorous lineages provided qualitatively similar results, therefore, we present results using the whole dataset. We identified an independent origin of myrmecochory when myrmecochorous species were not found in either (i) the sister group of the myrmecochorous lineage or (ii) the outgroup for the monophyletic group that included the myrmecochorous lineage and its sister group.

Next, we identified the phylogenetic sister group of each myrmecochorous lineage. To designate sister groups, we considered those phylogenetic reconstructions that were based on the most comprehensive data available in the study (consensus trees). We accepted a sister group only if its monophyly with the myrmecochorous lineage was established in the most comprehensive tree (bootstrap values >50%). We excluded comparisons in which outgroup analysis indicated that elaiosomes were lost in the non-myrmecochorous lineage. For plant nomenclature, we followed the Angiosperm Phylogeny Group (APG, version 9; as implemented in [44]).

For the analyses presented here, we excluded genera (i) where many species have elaiosomes, but dispersal mode could not be reliably determined for most species (e.g., *Acalypha*, *Arenaria*, *Carduus*, *Carex*, *Cirsium*, *Jatropha*, *Peperomia* etc.), (ii) where seed dispersal by ants is part of a more general mutualism between ants and plants (ant-garden epiphytes *Aechmea*, *Anthurium*, *Hoya*, *Nidularium*, *Philodendron* etc.), and (iii) where sister groups could not be reliably established due to a lack of adequately sampled phylogenetic trees (*Acacia*, *Calathea*, *Cleome*, *Globba*, *Iris*, *Primula*, *Potentilla* etc.). In total, our dataset for analysis included myrmecochorous plants in 241 genera (c. 2% of all plant genera), distributed in 101 lineages in 55 plant families (c. 12% of plant families; Stevens [44] (**Table S1** in Supporting Information).

### Species diversity, seed dispersal mode, biogeographic distribution

Data on global species diversity (number of species) were taken primarily from the phylogenetic studies, or from [44] or [45]. To estimate the ancestral mode of seed dispersal in myrmecochorous lineages, it was most parsimonious to assume that the dispersal mode in the non-myrmecochorous sister group was ancestral to both lineages. We distinguished seed dispersal modes based on literature sources or from seed morphology as (i) passive if the seed had no obvious structural modification regarding dispersal, (ii) water-dispersed if the structure of the seed enabled it to float on water or be washed away by rainfall, (iii) wind-dispersed if the seed had wings, plumes or hairs playing a role in airborne transport, (iv) externally vertebrate-dispersed (exozoochory) if the seeds had hooks, barbs or sticky substances that attach to feather and fur, or (v) internally vertebrate-dispersed (endozoochory) if the seed was surrounded by fleshy pulp or large, red/orange arils [46]–[48]. Data on the geographical distribution of genera were collected primarily from the phylogenetic studies, or from [44] or [45]. To classify groups into biogeographical distribution types, we primarily used Wallace's [49] biogeographical regions but designated other categories for more complex distributions (**Fig. 1B**).

### Sister-group comparisons

Sister-group comparisons offer a possibility to study replicated evolutionary events. Sister groups are, by definition, of the same evolutionary age [50], therefore, the difference in the number of lineages between two sister groups is due to differences in diversification rates rather than in lineage ages. Sister-group comparisons are typically used for testing “key innovations” in evolution, which are associated with higher diversification, e.g., [51], [52]. Although sister-group comparisons have been criticized based on the fact that they ignore information in tree topologies [53], the large taxonomical scale of this study and the lack of an adequate (genus-level) supertree for angiosperms precluded the use of methods based on tree topologies. Early methods to test key innovations using sister-group comparisons [54] have been shown to be biased by large differences in species diversity [55], and are prone to elevated Type I error rates [56], [57]. To avoid such biases, here we used sign-tests on species numbers and calculated a species diversity contrast to test differences between sister groups, as proposed by Mitter et al. [52], and as recommended by Vamosi & Vamosi [58] in their review of the methods for sister-group comparisons.

### Variables and analyses

We used the direction and magnitude of differences in species diversity between sister groups as response variables. First, we tallied the number of comparisons where the direction of the difference supported the hypothesis (myrmecochores>non-myrmecochores) and tested whether comparisons supporting the hypothesis were more frequent than expected by chance using a sign test [58]. Second, to characterize differences in magnitude, we calculated a species diversity contrast as *log(X)−log(Y)*, where *X* is species number in the myrmecochorous lineage and *Y* is species number in the sister group. This formula corrects for the exponential nature of diversification through the log-transformation [59], [60], is independent of the divergence time and total diversity of the two lineages [61], and can be easily interpreted as proportional differences (*log(X)−log(Y)* = *log(X/Y)*). We tested whether mean species diversity contrasts deviated from 0, which is expected under the null hypothesis of no difference between sister lineages, using a one-sample t-test. We also tested whether the direction or magnitude of the differences between myrmecochorous lineages and their sister groups were contingent on dispersal mode of the sister group or biogeographic region in two general linear mixed-effects models constructed in R [62]. Model 1 was a logistic regression (function ‘glmmPQL’) in which the binary response variable was whether a comparison supported the hypothesis or not. The response variable in Model 2 was species diversity contrast, which was a continuous variable (function ‘lme’). In both models, dispersal mode in the sister group (five levels) was the fixed effect and biogeographical distribution type (12 levels) was a random effect because the latter was a sample of an unknown number of potential distribution types [63]. The models were estimated by restricted maximum-likelihood, allowed the within-group errors to be correlated and/or have unequal variances, and were robust to unbalanced designs [62]. The influence of the random effect in both models was evaluated by comparing the intercept S.D. to the residual S.D., as recommended in [64]. Finally, we also repeated the tests of sister-group dispersal mode and biogeographic distribution type using a Bayesian fit of the models of the fixed and random effects. The results were qualitatively identical to the mixed-effects models reported in the text.

## Supporting Information

Table S1 Myrmecochorous plant lineages, their sister groups and data on seed dispersal mode in the sister-group, biogeographic distribution of lineages (M + N−M) and species number used in the analyses presented in the paper.
(0.66 MB DOC)
