# Supplementary material for: Ants Sow the Seeds of Global Diversification in Flowering Plants
Source: PLoS One. 2009 May 13;4(5):e5480. doi: 10.1371/journal.pone.0005480 (PMC2674952; doi:10.1371/journal.pone.0005480)
Supplement: Table S1 — Myrmecochorous plant lineages, their sister groups and data on seed dispersal mode in the sister-group, biogeographic distribution of lineages (M + N−M) and species number used in the analyses presented in the paper. (0.66 MB DOC) [file pone.0005480.s001.doc]

**Online supporting information**

to accompany *Lengyel et al.* “Ants sow the seeds of global diversification in flowering plants”

**Table 1.** Myrmecochorous plant lineages, their sister groups and data on seed dispersal mode in the sister-group, biogeographic distribution of lineages (M + N-M) and species number used in the analyses presented in the paper.

| **No.** | **Family** | **Myrmecochorous lineage (M)** | **Sister group (N-M)** | **N-M dispersal mode** | **Biogeogr. distr. ^a^** | **No. species** | | **Myrmecochory reference** | **Phylogeny reference** |
| --- | --- | --- | --- | --- | --- | --- | --- | --- | --- |
|  |  |  |  |  |  | **M** | **N-M** |  |  |
| 1 | Aizoaceae | *Gunniopsis* | *Tetragonia* | Wind | HT | 15 | 57 | [1] | [2] |
| 2 | Aizoaceae | *Sesuvium, Trianthema* | *Tribulocarpus* | Water | WW | 29 | 1 | [1] | [2] |
| 3 | Amaryllidaceae | *Leucojum, Galanthus, Narcissus, Sternbergia, Vagaria, Lapiedra, Hannonia, Pancratium* | *Lycoris, Ungernia* | Wind | HA | 289 | 26 | [3-5] | [6] |
| 4 | Apiaceae | *Platysace, Xanthosia, Actinotus* | *Centella, Micropleura, Mackinlaya, Apiopetalum* | Water | PnT | 68 | 58 | [3,7] | [8] |
| 5 | Aristolochiaceae | *Asarum, Saruma* | *Aristolochia, Lactoris* | Wind | WW | 86 | 406 | [3,9-11] | [12,13] |
| 6 | Asteraceae | *Amberboa, Volutaria, Mantisalca, Cyanopsis, Goniocaulon, Plagiobasis, Karvandarina, Russowia, Tricholepis* | *Klasea, Serratula, Acroptilon, Oligochaeta, Leuzea* | Wind | PA | 107 | 99 | [14] | [15-17] |
| 7 | Asteraceae | *Centaurea* | *Zoegea, Stizolophus* | Wind | PA | 383 | 17 | [4,14,18-20] | [15] |
| 8 | Asteraceae | *Chrysogonum* | *Berlandiera* | Wind | NA | 18 | 11 | [14] | [21] |
| 9 | Asteraceae | *Cullumia* | *Berkheya, Cuspidia, Heterorachis, Didelta* | Exozoochory | PT | 26 | 99 | [22] | [23] |
| 10 | Asteraceae | *Dymondia* | *Arctotis, Arctotheca, Haplocarpha, Cymbonotus* | Wind | PT | 1 | 78 | [22] | [24] |
| 11 | Asteraceae | *Euryops* | *Cineraria, Dendrosenecio* | Wind | PT | 100 | 62 | [22] | [25] |
| 12 | Asteraceae | *Galactites* | *Ptilostemon* | Wind | PA | 12 | 23 | [14] | [26] |
| 13 | Asteraceae | *Osmitopsis* | *Athanasia* | Wind | PT | 11 | 54 | [22] | [27] |
| 14 | Asteraceae | *Osteospermum* | *Calendula* | Wind | OW | 153 | 20 | [22] | [27] |
| 15 | Asteraceae | *Wedelia* | *Elaphandra* | Passive | NW | 100 | 16 | [22,28] | [29] |
| 16 | Berberidaceae | *Epimedium, Vancouveria*, *Bongardia* | *Podophyllum, Sinopodophyllum, Diphylleia, Dysosma, Achlys* | Endozoochory | HA | 67 | 21 | [14,30-32] | [33-35] |
| 17 | Berberidaceae | *Gymnospermium* | *Leontice* | Endozoochory | HA | 12 | 28 | [14] | [33-35] |
| 18 | Boraginaceae | *Nemophila* | *Pholistoma* | Water | NA | 11 | 3 | [4,14,30,36] | [37] |
| 19 | Bruniaceae | *Audouinia capitata* | *Tittmannia* | Passive | PT | 1 | 25 | [22] | [38] |
| 20 | Bruniaceae | *Lonchostoma* | *Mniothamnea, Raspalia* | Passive | PT | 10 | 4 | [22] | [38] |
| 21 | Buxaceae | *Buxus, Notobuxus* | *Sarcococca, Pachysandra, Styloceras* | Endozoochory | WW | 90 | 20 | [14,39] | [40] |
| 22 | Cactaceae | *Aztekium* | *Echinocactus, Astrophytum* | Exozoochory | NW | 2 | 10 | [41] | [42] |
| 23 | Cactaceae | *Gymnocalycium* | *Browningia, Cereus, Coleocephalocereus, Micranthocereus, Samaipaticereus, Echinopsis, Harrisia, Haageocereus, Matucana, Oreocereus, Rauhocereus* | Exozoochory | NW | 70 | 1100 | [41] | [42] |
| 24 | Caricaceae | *Carica* | *Moringa* | Wind | PnT | 23 | 12 | [14] | [43] |
| 25 | Caryophyllaceae | *Moehringia* | *Arenaria* subg. *Arenaria,* subg. *Leiosperma, ‘Pseudomoehringia’* group | Wind | HA | 31 | 172 | [4,14,44] | [45] |
| 26 | Colchicaceae | *Colchicum, Androcymbium, Hexacyrtis, Ornithoglossum, Sandersonia, Gloriosa, Baeometra, Wurmbea, Camptorrhiza, Iphigenia, Schelhammera, Tripladenia, Disporum, Uvularia, Kuntheria,* | *Burchardia* | Passive | WW | 245 | 5 | [3,7,10,46-48] | [49] |
| 27 | Dasypogonaceae | *Dasypogon* | *Calectasia* | Passive | AU | 4 | 11 | [47] | [50] |
| 28 | Dilleniaceae | *Hibbertia, Adrastaea, Pachynema* | *Dillenia* | Endozoochory | PnT | 124 | 60 | [3,7] | [51] |
| 29 | Dipsacaceae | *Knautia* | *Pterocephalidium* | Wind | PA | 60 | 1 | [10,32,52] | [53] |
| 30 | Dipsacaceae | *Scabiosa* | *Sixalix* | Wind | PA | 80 | 10 | [14] | [53] |
| 31 | Elaeocarpaceae | *Tetratheca* | *Platytheca* | Water | AU | 39 | 2 | [3,7,54,55] | [54,56] |
| 32 | Euphorbiaceae | *Astraea* | *Ophellantha, Acidocroton* | Endozoochory | NT | 10 | 12 | [57] | [57] |
| 33 | Euphorbiaceae | *Bertya, Beyeria, Ricinocarpos* | *Baloghia, Fontainea* | Endozoochory | AU | 45 | 29 | [3,7,14] | [58] |
| 34 | Euphorbiaceae | *Claoxylon* | *Erythrococca* | Endozoochory | OW | 75 | 50 | [7] | [58,59] |
| 35 | Euphorbiaceae | *Conceveiba* | *Gavarretia, Polyandra* | Passive | NT | 18 | 2 | [60] | [58] |
| 36 | Euphorbiaceae | *Euphorbia, Chamaesyce, Synadenium, Monadenium, Pedilanthus, Neoguillauminia, Calycopeplus, Anthosterna, Dichostemma* | *Spirostachys, Excoecaria, Sebastiana, Colliguaja, Sapium, Stillingia, Adenopeltis, Grimmeodendron, Bonania, Hippomane, Ophthalmoblapton, Tetraplandra, Pachystroma, Hura, Gymnanthes, Mabea, Microstachys, Homalanthus, Neoshirakia, Dalembertia, Triadica, Actinostemon, Pseudosenefeldera, Senefelderopsis, Maprounea* | Endozoochory | WW | 1846 | 407 | [3,4,61,62] | [58] |
| 37 | Euphorbiaceae | *Monotaxis, Adriana, Amperea* | *Cephalomappa, Koilodepas, Cephalocroton, Sumbaviopsis, Melanolepis, Chrozophora, Discocleidion, Ricinus, Speranskia* | Passive | OW | 31 | 37 | [3,60] | [58] |
| 38 | Euphorbiaceae | *Pera, Clutia, Chaetocarpus* | *Pogonophora* | Wind | PnT | 108 | 6 | [22,58,60] | [58] |
| 39 | Euphorbiaceae | *Seidelia* | *Leidesia* | Passive | PT | 4 | 1 | [60] | [58] |
| 40 | Fabaceae | *Cytisus* | *Cytisophyllum, Laburnum* | Wind | PA | 60 | 3 | [63,64] | [65,66] |
| 41 | Fabaceae | *Daviesia, Viminaria, Erichsenia, Goodia, Bossiaea, Platylobium, Muelleranthus, Ptychosema, Aenictophyton* | *Gompholobium, Sphaerolobium* | Passive | AU | 287 | 43 | [3,7,67] | [68] |
| 42 | Fabaceae | *Hardenbergia, Kennedia* | *Mucuna, Desmodium, Lespedeza, Kummerowia, Campylotropis* | Water | PnT | 59 | 500 | [3,69,70] | [71] |
| 43 | Fabaceae | *Hovea, Templetonia, Lamprolobium* | *Brongniartia, Plagiocarpus* | Wind | HT | 52 | 57 | [3,7] | [72] |
| 44 | Fabaceae | *Pultenaea (= Mirbelia s. l.)* | *Isotropis* | Passive | AU | 470 | 16 | [3,7,70,73] | [68,74] |
| 45 | Fabaceae | *Ulex, Stauracanthus* | *Genista sect. Cephalospartum* | Passive | HA | 21 | 5 | [4,18,52] | [66] |
| 46 | Gesneriaceae | *Chrysothemis* | *Nautilocalyx* | Endozoochory | NT | 12 | 55 | [32,75] | [76] |
| 47 | Gesneriaceae | *Codonanthe* | *Nematanthus* | Endozoochory | NT | 34 | 48 | [14] | [76] |
| 48 | Goodeniaceae | *Dampiera* | *Anthotium* | Wind | AU | 66 | 5 | [3,7] | [77] |
| 49 | Goodeniaceae | *Goodenia, Scaevola, Verreauxia, Velleia, Coopernookia* | *Brunonia* | Passive | AU | 350 | 3 | [3,7,69] | [77] |
| 50 | Gyrostemonaceae | *Gyrostemon, Codonocarpus, Walteranthus, Tersonia, Reseda* | *Tovaria* | Passive | AU | 18 | 2 | [3] | [43,78] |
| 51 | Hemerocallidaceae | *Caesia, Hensmania, Johnsonia, Arnocrinum* | *Tricoryne* | Exozoochory | AU | 58 | 12 | [3,5,7] | [50] |
| 52 | Hyacinthaceae | *Lachenalia* | *Polyxena, Periboea* | Passive | PT | 110 | 23 | [14] | [79] |
| 53 | Hyacinthaceae | *Scilla, Chionodoxa* | *Bellevalia* | Wind | PA | 40 | 50 | [4] | [80] |
| 54 | Iridaceae | *Witsenia, Klattia* | *Nivenia* | Passive | PT | 4 | 10 | [81] | [82,83] |
| 55 | Juncaceae | *Luzula* | *Juncus, Distichia, Marsippospermum, Rostkovia* | Water | WW | 115 | 249 | [3,9,20] | [84] |
| 56 | Lamiaceae | *Ajuga* | *Caryopteris, Trichostema* | Passive | WW | 50 | 37 | [3,85] | [86] |
| 57 | Lamiaceae | *Lamium* | *Marrubium* | Exozoochory | HA | 190 | 139 | [4,14] | [86] |
| 58 | Lamiaceae | *Rosmarinus* | *Perovskia* | Wind | PA | 22 | 10 | [14,85] | [87] |
| 59 | Lamiaceae | *Teucrium* | *Teucridium, Oncinocalyx* | Exozoochory | WW | 250 | 2 | [14] | [86] |
| 60 | Liliaceae | *Erythronium* spp. - Eurasian + Eastern North American lineage | *Erythronium* spp. - Western North American lineage | Passive | HA | 8 | 17 | [5,88,89] | [90] |
| 61 | Liliaceae | *Gagea* | *Lloydia* | Passive | HA | 91 | 12 | [5,88] | [50,88,91] |
| 62 | Liliaceae | *Scoliopus* | *Streptopus* | Endozoochory | HA | 5 | 30 | [14,88,92] | [88] |
| 63 | Limeaceae | *Macarthuria* | *Limeum* | Endozoochory | OW | 12 | 21 | [3,73] | [93] |
| 64 | Malvaceae | *Gossypium* sect. *Grandicalyx* ('genome K' lineage) | *Gossypium* sect. *Sturtia* ('genom C'), sect. *Hibiscoidea* ('genome G') | Water | AU | 12 | 5 | [3,7] | [94,95] |
| 65 | Malvaceae | *Lasiopetalum, Hannafordia, Maxwellia, Thomasia, Guichenotia, Commersonia, Rulingia, Keraudrenia, Seringia* | *Hermannia, Waltheria, Melochia* | Endozoochory | WW | 167 | 205 | [3,7,70] | [96] |
| 66 | Melanthiaceae | *Trillium* | *Paris, Daiswa, Kinugasa, Trillidium* | Endozoochory | HA | 38 | 27 | [5,9,14] | [97] |
| 67 | Papaveraceae | *Corydalis, Dicentra, Adlumia, Dactylocapnos, Rupicapnos, Pseudofumaria, Cysticapnos* | *Hypecoum* | Exozoochory | HA | 384 | 18 | [9,22] | [98] |
| 68 | Papaveraceae | *Sanguinaria, Chelidonium, Eomecon, Macleaya, Bocconia, Hylomecon, Stylophorum* | *Dicranostigma* | Passive | HA | 99 | 8 | [4,9,10,14] | [99] |
| 69 | Penaeaceae | *Penaea, Brachysiphon, Endonema, Saltera, Stylapterus, Sonderothamnus* | *Olinia* | Endozoochory | PT | 23 | 8 | [22] | [100-102] |
| 70 | Phyllanthaceae | *Breynia* | *Sauropus* | Passive | IM | 35 | 40 | [3,7] | [103] |
| 71 | Picrodendraceae | *Picrodendron, Micrantheum, Oldfieldia, Stachystemon, Aristogeitonia, Scagea, Tetracoccus* | *Podocalyx, Paradrypetes* | Passive | WW | 82 | 3 | [3,7,58,62,104] | [105], Wurdack unpubl. from [58] |
| 72 | Poaceae | *Chionachne* | *Phacelurus* | Wind | IM | 9 | 10 | [32] | [106] |
| 73 | Poaceae | *Melica* | *Glyceria* | Water | HA | 80 | 35 | [4,14,30] | [107] |
| 74 | Polygalaceae | *Polygala, Bredemeyera, Muraltia, Nylandtia, Heterosamara, Salomonia, Comesperma, Monnina, Securidaca* | *Carpolobia, Atroxima* | Endozoochory | WW | 910 | 6 | [3,7,14,108] | [108] |
| 75 | Portulacaceae | *Claytonia, Montia* | *Lewisia* | Wind | NW | 41 | 16 | [9,14,32] | [109,110] |
| 76 | Primulaceae | *Cyclamen* | *Coris* | Exozoochory | PA | 20 | 3 | [14,32] | [111] |
| 77 | Proteaceae | *Grevillea* | *Buckinghamia, Opisthiolepis* | Wind | AU | 260 | 3 | [3,7,112] | [113,114] |
| 78 | Proteaceae | *Mimetes, Orothamnus, Leucospermum, Diastella, Sorocephalus, Spatalla, Paranomus, Vexatorella, Serruria, Leucadendron, Adenanthos* | *Isopogon* | Wind | HT | 308 | 65 | [3,115] | [113,114,116] |
| 79 | Ranunculaceae | *Anemone* | *Pulsatilla, Knowltonia* | Wind | HA | 120 | 48 | [4,9,14] | [117] |
| 80 | Ranunculaceae | *Delphinium* | *Consolida* | Wind | HA | 250 | 40 | [14] | [117] |
| 81 | Ranunculaceae | *Ficaria* | *Coptidium* | Passive | PA | 5 | 2 | [118] | [119] |
| 82 | Ranunculaceae | *Helleborus* | *Myosurus, Nigella, Actaea, Cimicifuga* | Passive | HA | 20 | 54 | [4,14,32,120] | [117] |
| 83 | Ranunculaceae | *Trollius, Adonis* | *Caltha* | Water | HA | 55 | 10 | [14] | [117] |
| 84 | Restionaceae | *Restio* | *Elegia, Chondropetalum, Dovea* | Wind | PT | 88 | 73 | [3,7] | [84,121] |
| 85 | Rhamnaceae | *Phylica, Trichocephalus* | *Nesiota, Noltea* | Endozoochory | PT | 151 | 2 | [22] | [122] |
| 86 | Rhamnaceae | *Pomaderris, Spyridium, Trymalium, Siegfriedia, Cryptandra, Stenanthemum* | *Colletia, Discaria, Alphitonia, Granitites* | Passive | HT | 172 | 50 | [3,7,54,69] | [122] |
| 87 | Rubiaceae | *Opercularia, Pomax* | *Leptostigma, Durlingtonia, Nertera, Normandia, Coprosma* | Endozoochory | HT | 19 | 112 | [7] | [123,124] |
| 88 | Rutaceae | *Asterolasia* | *Drummondita, Muiriantha* | Endozoochory | AU | 21 | 9 | [125] | [126] |
| 89 | Rutaceae | *Phebalium, Microcybe* | *Nematolepis, Rhadinothamnus, Chorilaena* | Passive | AU | 31 | 11 | [3,7,125] | [126] |
| 90 | Santalaceae | *Thesium, Osyridicarpos* | *Buckleya* | Endozoochory | WW | 177 | 7 | [52,127] | [128] |
| 91 | Sapindaceae | *Cardiospermum* | *Paullinia* | Endozoochory | PnT | 61 | 195 | [129] | [130] |
| 92 | Sapindaceae | *Dodonaea* | *Distichostemon* | Passive | HT | 70 | 8 | [3,131,132] | [130] |
| 93 | Scrophulariaceae | *Melampyrum* | *Bartsia, Lathraea, Rhinanthus, Rhynchocorys, Parentucellia, Odontites, Euphrasia, Tozzia* | Wind | HA | 35 | 310 | [4,14,30,118,133] | [134,135] |
| 94 | Scrophulariaceae | *Pedicularis* | *Agalinis, Esterhazya, Aureolaria, Seymeria, Lamourouxia, Castilleja, Orthocarpus, Cordylanthus, Triphysaria* | Wind | HA | 350 | 341 | [4,14,30,133] | [134,135] |
| 95 | Solanaceae | *Datura* | *Brugmansia* | Passive | NW | 11 | 5 | [14] | [136] |
| 96 | Tecophilaeaceae | *Cyanastrum* | *Walleria* | Passive | PT | 7 | 3 | [14] | [82] |
| 97 | Turneraceae | *Turnera* | *Piriqueta* | Passive | NT | 100 | 44 | [137,138] | [139] |
| 98 | Urticaceae | *Parietaria* | *Boehmeria* | Exozoochory | WW | 10 | 80 | [10,32] | [140] |
| 99 | Valerianaceae | *Fedia* | *Valerianella* | Wind | PA | 3 | 50 | [14] | [141] |
| 100 | Violaceae | *Viola* | *Noisettia* | Passive | WW | 400 | 8 | [3,4,7,9,10,20] | [142] |
| 101 | Zygophyllaceae | *Zygophyllum, Augea, Fagonia* | *Larrea, Pintoa, Bulnesia, Porlieria, Guaiacum* | Wind | WW | 120 | 31 | [22] | [143,144] |

^a^ Biogeographic distribution types for lineages comprising both M and N-M lineages:

AU – Australian, IM – Indo-Malayan, NA – Nearctic, NT – Neotropical, PA – Palearctic, PT – Paleotropical (mostly Cape Floristic Region); complex distribution types: HA – Holarctic (NA + PA), NW – New World (NA + NT), OW – Old World (AU + IM + PA + PT), PnT – Pan-tropical (AU + IM + NT + PT), HT – Holotropical (AU + NT + PT), WW – Worldwide.

**References**
